# Astaxanthin Inhibits Autophagic Cell Death Induced by Bisphenol A in Human Dermal Fibroblasts

**DOI:** 10.3390/antiox10081273

**Published:** 2021-08-11

**Authors:** Seong-Ryeong Lim, Do-Wan Kim, Junghee Sung, Tae Hoon Kim, Chang-Hyung Choi, Sei-Jung Lee

**Affiliations:** 1 Department of Pharmaceutical Engineering, Daegu Haany University, Gyeongsan 38610, Korea; mang9811@dhu.ac.kr (S.-R.L.); sosr200211@dhu.ac.kr (D.-W.K.); 2 Research Center, Reanzen Co., Ltd., Anyang 14056, Korea; jhsung@reanzen.com; 3 FoodyWorm Inc., Yancheongsongdae-gil 10, Ochang-eup, Cheongwon-gu, Choenju-si 28118, Korea; ceo@foodyworm.com; 4 Division of Cosmetic Science and Technology, Daegu Haany University, Gyeongsan 38610, Korea

**Keywords:** astaxanthin, autophagic cell death, bisphenol A, normal human dermal fibroblasts, reactive oxygen species

## Abstract

Astaxanthin, a natural antioxidant carotenoid, is a nutrient with diverse health benefits, given that it decreases the risk of oxidative stress-related diseases. In the present study, we investigate the functional role of astaxanthin during autophagic cell death induced by the estrogenic endocrine-disrupting chemical bisphenol A (BPA) in normal human dermal fibroblasts (NHDF). BPA significantly induced apoptotic cell death and autophagy in NHDF. Autophagic cell death evoked by BPA was significantly restored upon a treatment with astaxanthin (10 μM) via the inhibition of intracellular reactive oxygen species (ROS) production. Astaxanthin inhibited the phosphorylation of extracellular signal-regulated kinases (ERK) stimulated by ROS production, but it did not influence the activation of c-Jun N-terminal kinase (JNK) and p38 mitogen-activated protein kinase (MAPK) in BPA-treated NHDF. Astaxanthin abrogated the ERK-mediated activation of nuclear factor-kappa B (NF-κB), which is responsible for the mRNA expression of LC3-II, Beclin-1, Atg12, and Atg14 during apoptotic cell death induced by BPA. These results indicate that astaxanthin is a pharmacological and nutritional agent that blocks the skin fibroblastic autophagic cell death induced by BPA in human dermal fibroblasts.

## 1. Introduction

Bisphenol A (BPA), ubiquitously present in consumer products containing polycarbonate plastics and epoxy resins, has been utilized extensively in dentistry, food packaging, thermal printing papers, and lacquers [1]. Owing to its hormone-like features, BPA is known to manipulate the activation of many estrogen receptors in mammalian cells, evoking toxicological cellular responses related to prostate and breast cancer, as well as respiratory diseases [2,3]. While oral intake from contaminated food and drinks represents the predominant source of BPA exposure and given the well-known pathophysiological role of ingested BPA, the potential risk of BPA exposure via dermal contact and absorption in the skin is not well documented. Skin is the largest organ of human body consisting of three primary layers and diverse cell types [4], where dermal fibroblasts are an essential component, maintaining the structure of fibers by producing a collagen-rich extracellular matrix and playing an important role in the production of inflammatory mediators against chemical, microbial, viral, and fungal agents [5,6,7]. Given that approximately 46% of BPA is diffused through the skin [8], recent studies have suggested that BPA causes oxidative dermal damage and thus stimulates the expression of many pro-inflammatory mediators associated with human skin diseases [9]. However, the underlying pathophysiological mechanisms of BPA involved in oxidative dermal damage remain undescribed.

BPA provokes oxidative stress that is caused by an excessive production of reactive oxygen species (ROS), resulting in damages to lipids, proteins, DNA, and organelles, and triggering to the induction of cellular dermal damage, which is the fundamental mechanisms mediating apoptosis and autophagy in skin diseases [10,11]. A recent study suggested that apoptosis and autophagy are the most common consequences of accumulative changes in the promoting of skin pathophysiology including the aberrant pigmentation, increased wrinkles, and telangiectasia [11]. Indeed, it was proven that excessive or uncontrolled levels of autophagy are closely related to the induction of cell death [12]. Given that the major role of oxidative stress induced by BPA is to promote cellular dermal damages, it is crucial to investigate pharmacological substances that regulate the apoptotic and autophagic pathway mediated by ROS in dermal fibroblasts.

Astaxanthin, a natural carotenoid found in marine organisms, is a well-known antioxidant classified as xanthophyll [13,14]. Many previous reports have shown that astaxanthin has a variety of pharmacological functions in many disease models, such as lung fibrosis [15], ischemia and reperfusion (I/R) injury [16], neuronal toxicity [17,18], and hematopoietic system injury [19], suggesting that astaxanthin has various health benefits in different systems of the body carrying out the different functions. Although astaxanthin has been receiving more attention due to its various biological functions, it is difficult to deliver to organs effectively as a result of the poor water solubility, hydrophobic/lipophilic characters, high melting point, and chemical instability of astaxanthin [20], suggesting that the unique structure of astaxanthin is one of the major factors limiting its oral and intraperitoneal bioavailability to develop as the therapeutic, medicinal, and food agents. Thus, it seems reasonable that the topical application of astaxanthin as a cosmetic source to the skin would prove much more effective in ameliorating the oxidative skin dermal damage [21,22]. However, the functional role of astaxanthin in oxidative skin dermal damage caused by BPA exposure had not yet been explored.

In this study, therefore, we investigate the pharmacological role of astaxanthin during apoptosis and autophagy elicited by BPA in human dermal fibroblasts and the mechanism underlying the beneficial effects of astaxanthin with regard to skin health.

## 2. Materials and Methods

### 2.1. Materials

RPMI-1640, streptomycin, penicillin, and fetal bovine serum (FBS) were obtained from GE Healthcare (Logan, UT, USA). Bisphenol A (BPA) and astaxanthin were from Sigma-Aldrich (St. Louis, MO, USA). JNK, ERK, p38 MAPK, NF-κBp65, p-JNK, p-ERK, p-p38 MAPK, p-NF-κBp65, Beclin-1, β-actin, and LC3-II antibodies were obtained from Santa Cruz Biotechnology (Paso Robles, CA, USA). HRP goat anti-mouse and -rabbit IgG antibodies were purchased from Abcam (Cambridge, MA, USA). Bay 11-7082, N-acetylcysteine (NAC), and PD98059 were from Tocris (Minneapolis, MN, USA). CM-H_2_DCFDA was purchased from Invitrogen (Carlsbad, CA, USA).

### 2.2. Cells

Normal human dermal fibroblasts (NHDF), obtained from the American Type Culture Collection (ATCC, Manassas, VA, USA), were grown in RPMI-1640 containing 10% fetal bovine serum, 100 U/mL penicillin, and 100 μg/mL streptomycin, and were cultured in a dish in a 37 °C incubator with humidified atmosphere of 5% CO_2_.

### 2.3. Inhibitor Treatment

Upon reaching ~80% confluency, NHDF were grown with serum-free media for 12 h and were then incubated with pharmacological inhibitors for NF-κB (Bay 11-7082, 1 μM), ROS (NAC, 1 μM), and ERK (PD98059, 1 μM) for 30 min prior to the BPA (50 μM) treatment. No inhibitors at those particular concentrations showed effects on cell viability of NHDF by themselves.

### 2.4. Cell Viability Assay

The effect of BPA and astaxanthin on cell viability of NHDF was evaluated by EZ-CYTOX kit (Dail-Lab Service, Seoul, Korea) as previously demonstrated [23], according to the manufacturer’s instructions. NHDF were incubated with the EZ-CYTOX master mix (10 μL) for 2 h. Cell viability was determined by using a microplate reader (SPARK, Seestrasse, Männedorf, Switzerland) at 450 nm.

### 2.5. Quantitative Real-Time Polymerase Chain Reaction (qRT-PCR)

Cellular RNA of NHDF was isolated and purified using the NucleoSpin^®^ RNA kit (Macherey-Nagel, Düren, Germany). Reverse transcription for the cDNA preparation was performed with total RNA by using a ReverTra Ace^®^ qPCR RT Master Mix (cDNA kit) (TOYOBO, Osaka, Japan). The autophagic genes responsible for the induction (ULK1/2, Vps34, Atg14, Beclin-1), expansion/closure (Atg5, Atg12, Atg16L1, LC-3II), and fusion/degradation (Rab7, FYCO1, LAMP1/2) in an autophagy pathway induced by BPA were amplified using a LightCycler 96 system (Roche, Basel, Switzerland) with a AccuPower^®^ 2X Greenstar qPCR Master Mix (Bioneer, Daejeon, Korea). The sequences for primer pairs used are described in Supplementary Materials Table S1.

### 2.6. Detection of Intracellular Reactive Oxygen Species (ROS)

NHDF was incubated with 10 mM of CM-H_2_DCFDA for 1 h to measure the level of intracellular ROS. After washes with PBS, cells were scraped and loaded into a black 96-well plate. The fluorescence, which corresponds to the amount of intracellular ROS, was determined using a microplate reader designed for the detection of fluorescent and luminescent signals (SPARK, Seestrasse, Männedorf, Switzerland) with excitation/emission at 485/535 nm.

### 2.7. Western Blot Analysis

Cellular protein of NHDF was extracted with RIPA lysis buffer (ATTO Corp., Tokyo, Japan) and the concentrations were determined by BCA protein assay kits (Pierce, Rockford, IL, USA). Western blot analysis was performed as previously described [23]. The band intensity of the western blot was calculated by using the Scion Image software (Scion Image Beta 4.02, Frederick, MD, USA).

### 2.8. Flow Cytometry

Apoptotic cells were detected with Annexin V-FITC Apoptosis Kit (BD Biosciences, Franklin Lakes, NJ, USA) and quantified the proportion of healthy, early apoptotic, late apoptotic, and necrotic cells by using the flow cytometry (Beckman Coulter, Fullerton, CA, USA) as previously described [24].

### 2.9. Statistical Analysis

Data are represented as mean value ± standard errors (S.E.). Statistical significance was analyzed by one-way analysis of variance (ANOVA) in SPSS 16 software (IBM Corp, Armonk, NY, USA). *p* < 0.05 is considered significant.

## 3. Results

### 3.1. Inhibitory Effect of Astaxanthin on the Dermal Cell Death and Autophagy Induced by BPA

**Figure 1:**
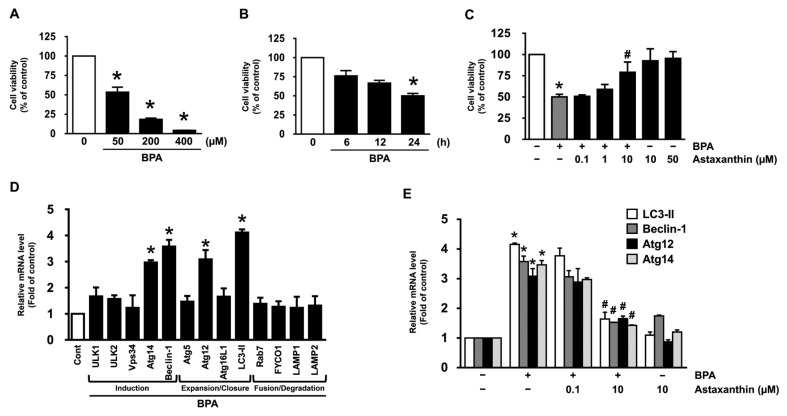
Inhibitory effect of astaxanthin on the dermal cell death and autophagy induced by BPA. (**A**) Dose responses of cell viability in normal human dermal fibroblasts (NHDF) treated with 50, 200, and 400 μM of BPA for 24 h are shown. * *p* ≤ 0.05 versus control. *n* = 3. (**B**) Time responses of cell viability treated with 50 μM of BPA for 24 h are shown. * *p* ≤ 0.001 versus control. *n* = 3. (**C**) NHDF was exposed to the BPA with astaxanthin (0.1, 1, and 10 μM) for 24 h. * *p* ≤ 0.001 versus control. # *p* ≤ 0.05 versus BPA alone. *n* = 3. (**D**) NHDF was treated with BPA for 24 h. The effect of BPA on the expression of autophagy-related genes was evaluated by qRT-PCR. * *p* ≤ 0.05 versus control. *n* = 3. (**E**) NHDF was exposed to the BPA in the presence of astaxanthin (0.1 and 10 μM) for 24 h. The mRNA level of the autophagic genes quantified by qRT-PCR is shown. * *p* ≤ 0.01 versus control. # *p* ≤ 0.01 versus BPA alone. *n* = 3.

To confirm that bisphenol A (BPA) induces the dermal cytotoxicity, normal human dermal fibroblasts (NHDF) were treated with BPA at various concentrations (0–400 μM) for 24 h. BPA markedly decreased the cell viability in a dose-dependent manner (Figure 1A). The cytotoxicity of NHDF also was observed from 24 h after treatment with 50 μM of BPA (Figure 1B). To determine the functional role of astaxanthin in the dermal fibroblastic damage, NHDF was exposed to the BPA in the presence astaxanthin at various concentrations (0.1–10 μM). A co-treatment with 10 μM of astaxanthin effectively abrogated the cytotoxicity evoked by BPA (Figure 1C). To know how BPA influences the autophagic process, we further evaluated the effect of BPA on the mRNA level of the autophagic genes responsible for the induction, expansion/closure, and fusion/degradation in an autophagy pathway. The prominent expressions of Atg14, Beclin-1, Atg12, and LC3-II responsible for the autophagic induction, expansion, and closure processes were observed following a BPA treatment (Figure 1D). However, the autophagy pathway initiated by BPA was significantly inhibited by treatment with astaxanthin at 10 μM (Figure 1E). These results indicate that the pharmacological effect of astaxanthin against BPA exposure in dermal fibroblast is related to the blocking of the cell death and autophagy.

### 3.2. Antioxidative Effect of Astaxanthin on the Production of ROS in NHDF Treated with BPA

**Figure 2:**
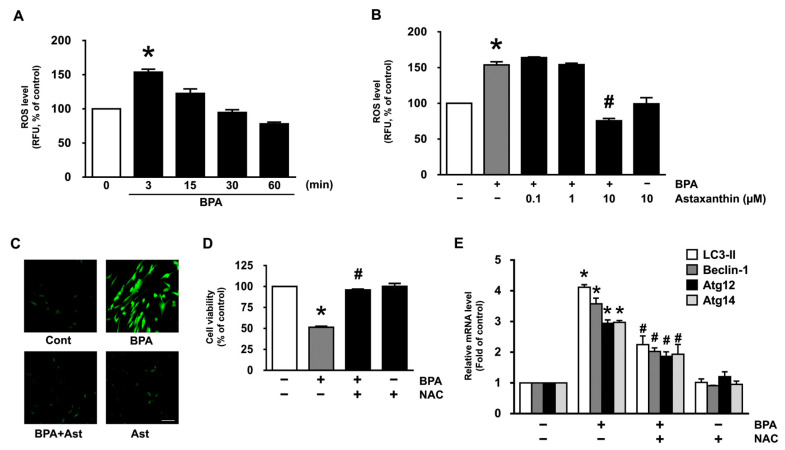
Antioxidative effect of astaxanthin on the production of ROS in NHDF treated with BPA. (**A**) Time responses of BPA (50 μM) on the ROS production determined by staining NHDF with CM-H_2_DCFDA are shown. * *p* ≤ 0.001 versus control. *n* = 3. RFU, relative fluorescence units. (**B**) NHDF was exposed to the BPA in the presence of astaxanthin (0.1 and 10 μM) for 3 min. * *p* ≤ 0.001 versus control. # *p* ≤ 0.001 versus BPA alone. *n* = 3. (**C**) The inhibitory effect of astaxanthin on ROS production induced by BPA (green) revealed by confocal microscopy is shown. Scale bars, 100 μm (magnification × 100). *n* = 3. (**D**,**E**) Cells were treated with 1 μM of N-acetylcysteine (NAC) as an antioxidant for 30 min prior to BPA exposure for 24 h. The effect of NAC on the levels of cell viability (**D**) and autophagic genes (**E**) is shown. * *p* ≤ 0.01 versus control. # *p* ≤ 0.05 versus BPA alone. *n* = 3.

BPA has been reported to evoke the cellular oxidative stress responsible for the production of reactive oxygen species (ROS) to amplify the signals for apoptosis and autophagy [10,11]. The prominent production of ROS was observed at 3 min following a BPA treatment (Figure 2A), though the production could be inhibited by astaxanthin at 10 μM (Figure 2B). The ROS scavenging effect of astaxanthin was further revealed by staining NHDF with a fluorescent dye, CM-H_2_DCFDA (Figure 2C). To ensure the production of ROS is involved in autophagy and cell death, NHDF was pretreated with an antioxidant, N-acetylcysteine (NAC) for 30 min prior to the BPA treatment. The levels of cytotoxicity and mRNA expressions of LC3-II, Beclin-1, Atg12, and Atg14 induced by BPA were significantly abrogated by the incubation with NAC (Figure 2D,E). These data suggest that the pharmacological effect of astaxanthin on cell death and autophagy in human dermal fibroblasts is mediated by its antioxidative potential against BPA.

### 3.3. Astaxanthin Inhibits ERK Activation Triggered by BPA

**Figure 3:**
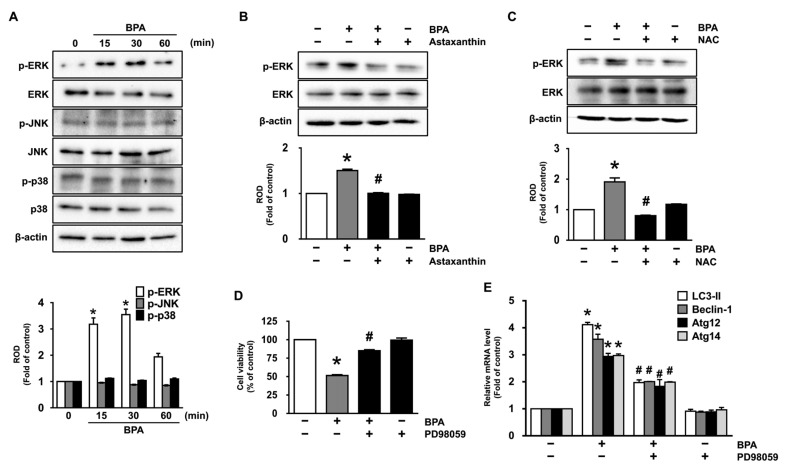
Astaxanthin inhibits ERK activation triggered by BPA. (**A**) Time responses of BPA (50 μM) on the phosphorylation of MAPKs are shown. * *p* ≤ 0.05 versus control. *n* = 3. ROD, relative optical density. (**B**) The role of astaxanthin in the activation of ERK is shown. * *p* ≤ 0.001 vs. control, # *p* ≤ 0.001 versus BPA alone. *n* = 3. (**C**) Cells were pretreated with NAC (1 μM) for 30 min prior exposure to BPA for 30 min. * *p* ≤ 0.001 vs. control. # *p* ≤ 0.001 vs. BPA alone. *n* = 3. (**D**,**E**) Cells were incubated with 1 μM of ERK inhibitor (PD98059) for 30 min prior to BPA exposure for 24 h. The effect of PD98059 on the levels of cell viability (**D**) and autophagic genes (**E**) is shown. * *p* ≤ 0.001 versus control. # *p* ≤ 0.01 versus BPA alone. *n* = 3.

We further examined the role BPA in the phosphorylation of mitogen-activated protein kinases (MAPKs), which are known as downstream mediators of ROS [25,26]. The phosphorylation of ERK was significantly increased for 15−30 min by treatment with BPA, while the JNK and p38 MAPK were not influenced by the BPA (Figure 3A). However, the ERK phosphorylation was significantly inhibited by treatment with 10 μM of astaxanthin (Figure 3B and Supplementary Materials Figure S1A) and antioxidant, NAC (Figure 3C), demonstrating that astaxanthin inhibits the ROS-mediated ERK activation induced by BPA. Moreover, we found that the ERK inhibitor (PD98059) significantly restored the dermal fibroblastic cytotoxicity and mRNA expression of autophagic genes in BPA-treated NHDF (Figure 3D,E). These data demonstrate that the ERK activation triggered by ROS is the critical step in the promotion of autophagic cell death caused by BPA and that the BPA signaling pathway in dermal fibroblast can be suppressed by astaxanthin treatment.

### 3.4. Inhibitory Effects of Astaxanthin on the Phosphorylation of NF-κB Stimulated by BPA

**Figure 4:**
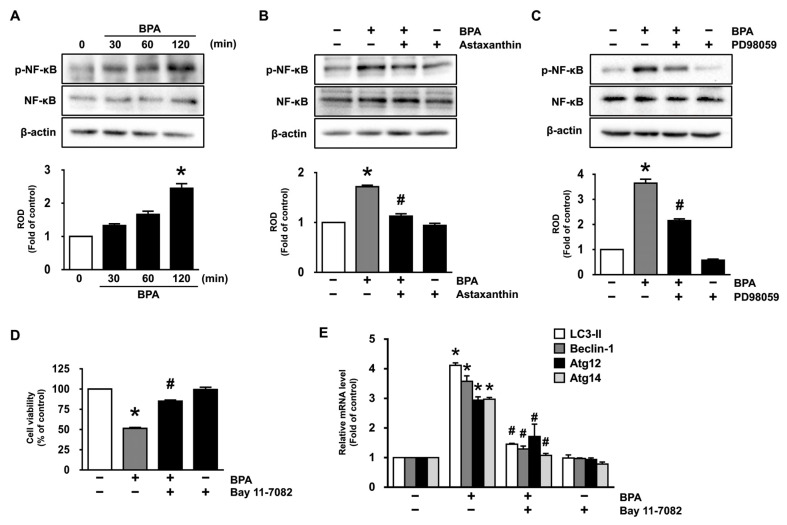
Inhibitory effects of astaxanthin on the phosphorylation of NF-κB stimulated by BPA. (**A**) Time responses of BPA (50 μM) on the phosphorylation of NF-κB are shown. * *p* ≤ 0.05 versus control. *n* = 3. ROD, relative optical density. (**B**) The role of astaxanthin in the activation of NF-κB is shown. * *p* ≤ 0.001 versus control, # *p* ≤ 0.001 versus BPA alone. *n* = 3. (**C**) Cells were incubated with 1 μM of PD98059 for 30 min prior to BPA exposure for 120 min. * *p* ≤ 0.05 versus control. # *p* ≤ 0.001 versus BPA alone. *n* = 3. (**D**,**E**) Cells were incubated with 1 μM of NF-κB inhibitor (Bay 11-7082) for 30 min prior to BPA exposure for 24 h. The effect of Bay 11-7082 on the levels of cell viability (**D**) and autophagic genes (**E**) is shown. * *p* ≤ 0.001 versus control. # *p* ≤ 0.001 versus BPA alone. *n* = 3.

Having demonstrated the necessity of ERK in the regulation of the dermal fibroblastic cytotoxicity and autophagy initiated by BPA, we have further checked the regulatory effect of ERK on the phosphorylation of nuclear factor-kappa B (NF-κB) that induces the transcriptional gene expression related to the autophagic cell death. BPA significantly induced the phosphorylation of NF-κB at 120 min (Figure 4A), though the increase could be blocked by treatment with 10 μM of astaxanthin (Figure 4B and Supplementary Materials Figure S1B). The transcriptional activation of NF-κB induced by BPA was markedly abrogated by treatment with the ERK inhibitor (PD98059) (Figure 4C), demonstrating that the ERK activation is required for the phosphorylation of NF-κB in the promoting of autophagic cell death caused by BPA. Moreover, a NF-κB inhibitor, Bay 11-7082, significantly inhibited the levels of dermal fibroblastic cytotoxicity and mRNA expression of autophagic genes induced by BPA (Figure 4D,E), indicating that the activation of NF-κB is involved in the autophagic cell death induced by BPA. Thus, these data demonstrate that astaxanthin negatively regulates the phosphorylation of NF-κB mediated by ERK, which is necessary for the autophagic cell death occurred by the BPA signaling pathway in dermal fibroblasts.

### 3.5. Astaxanthin Regulates the Autophagic Cell Death Triggered by BPA 

**Figure 5:**
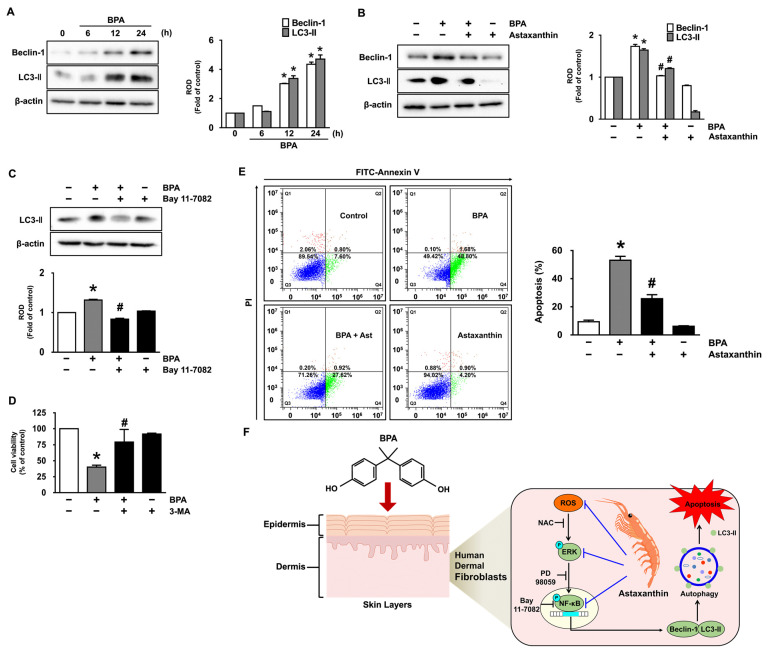
Astaxanthin regulates the autophagic cell death triggered by BPA (**A**) Time responses of BPA (50 μM) on the protein expression of Beclin-1 and LC3-II are shown. * *p* ≤ 0.001 versus 0 h. *n* = 3. ROD, relative optical density. (**B**) The role of astaxanthin in the expression of autophagy-related proteins is shown. * *p* ≤ 0.005 versus control, # *p* ≤ 0.001 versus BPA alone. *n* = 3. (**C**) Cells were incubated with 1 μM of Bay 11-7082 for 30 min prior to BPA exposure for 24 h. * *p* ≤ 0.001 versus control. # *p* ≤ 0.001 versus BPA alone. *n* = 3. (**D**) Cells were incubated with 1 μM of autophagy inhibitor (3-MA) for 30 min prior to BPA exposure for 24 h. The effect of 3-MA on the levels of cell viability is shown. * *p* ≤ 0.001 versus control. # *p* ≤ 0.01 versus BPA alone. *n* = 3. (**E**) The proportion of apoptotic cells stained by Annexin V was analyzed by performing flow cytometry. * *p* ≤ 0.05 versus control. # *p* ≤ 0.05 versus BPA alone. *n* = 3. (**F**) The sequences of putative signaling pathways are summarized.

We further tried to clarify whether astaxanthin regulates the protein expression of autophagy responsible for the autophagic cell death triggered by BPA. The levels of protein expression of Beclin-1 and LC3-II, as representative markers of autophagy activation, were investigated in NHDF treated with the BPA. BPA markedly augmented the protein expression of Beclin-1 and LC3-II in a time-dependent manner (Figure 5A). However, the prominent expression of the autophagy-related proteins elicited by BPA was significantly inhibited by treatment with 10 μM of astaxanthin (Figure 5B and Supplementary Materials Figure S1C). Interestingly, we found that the NF-κB inhibitor (Bay 11-7082) significantly suppressed the expression of LC3-II induced by BPA (Figure 5C), indicating that NF-κB phosphorylation enhances transcriptional expression of LC3-II during the autophagic cell death triggered by BPA. We next checked how activated autophagic process coordinates with the apoptotic cell death in BPA-treated NHDF by treating with an autophagy inhibitor, 3-methyladenine (3-MA). We found that 3-MA effectively restores the cell viability decreased by BPA (Figure 5D), indicating that the autophagy activation is required for the dermal fibroblastic damage caused by BPA.

To distinguish the aspects of cell death outcomes induced by BPA, we next undertook flow cytometric analyses by staining the dermal fibroblasts with an Annexin V/Propidium Iodide (PI). BPA significantly induced the apoptotic cell death of NHDF for 24 h, whereas for necrotic cell death, a marginal effect was noted. However, the BPA-induced apoptotic cell death was significantly decreased by treatment with astaxanthin (Figure 5E). These results indicate that the pharmacological effect of astaxanthin on human dermal fibroblasts is related to the blocking of apoptotic cell death caused by BPA.

## 4. Discussion

Our results suggest that BPA induces the dermal fibroblastic autophagy pathway, which is closely related to apoptotic cell death, and that astaxanthin neutralizes the autophagic cell death pathways initiated by BPA through the inhibition of ROS-mediated activation of ERK to suppress the phosphorylation of NF-κB responsible for the transcriptional expression of the autophagy genes (LC3-II, Beclin-1, Atg12, Atg14) in human dermal fibroblasts (Figure 5F). Thus, we suggest that one of new pharmacological mechanisms of astaxanthin that would inhibit the skin dermal damages promoted by BPA is to reduce the dermal fibroblastic autophagy together with apoptosis. To our knowledge, our results are the first report demonstrating the role of BPA in the control of the skin fibroblastic autophagic cell death as inhibited by astaxanthin.

Although a protective effect of astaxanthin against BPA in dermal fibroblasts has not been reported, many scientists have found that astaxanthin has many positive dermatological effects on human skin damage, aging, and inflammation via its antioxidant effect [27]. These results are consistent with our finding that astaxanthin has the ability to inhibit the oxidative autophagic cell death induced by BPA in NHDF. BPA is a chemical produced at among the highest volumes during the production of polycarbonate plastic and epoxy resin; it can stimulate the destruction of mitochondria, the endoplasmic reticulum, and lysosomes via oxidative damage, resulting in aberrant pigmentation, laxity, and wrinkle formation in the skin [1,11]. Moreover, BPA has been reported to stimulate the fibroblastic apoptosis by arresting the cell cycle as well as autophagy by upregulating the expression of autophagy-related protein during the promotion of childhood respiratory diseases [3]. Although proper physiological levels of autophagy are essential for the digestion of the cellular contents within lysosomes so as to maintain a proper level of skin homeostasis, excessive or uncontrolled levels of autophagy can induce autophagy-dependent apoptosis [12]. Hence, it is plausible that BPA amplifies dermal fibroblast autophagic cell death signals by producing intracellular ROS. Indeed, astaxanthin has been found to scavenge the skin ROS responsible for various aging manifestations, such as a loss of skin elasticity, wrinkle formation, and impaired wound healing [27]. These findings also therefore suggest that astaxanthin is a potential antioxidative prophylactic agent that can prevent oxidative stress-related diseases that affect human dermal fibroblasts when they are exposed to BPA.

To find the underlying molecular mechanisms related to how ROS participates in the initiation of the autophagic cell death pathways, we focused on mitogen-activated protein kinases (MAPKs), specifically ERKs, p38 MAPK, and JNKs, with regard to their possible roles in the regulation of the activity of many transcription factors and cellular enzymes and, thus, involvement in the biological responses of cells to external stimuli as downstream mediators of ROS [25,26]. Despite the functional roles of MAPKs governed by the ROS signaling pathway in the promotion of autophagy [25,26], we noted that astaxanthin has an inhibitory effect on the phosphorylation of ERK as uniquely stimulated by BPA. These results are similar to an earlier result showing that ERK activation is a necessary step in the promotion of apoptosis in hippocampal HT-22 cells exposed to the BPA [28], suggesting that ERK is an important signaling mediator of BPA during autophagic cell death in dermal fibroblasts. Moreover, we found convincing evidence that ROS production is required for ERK phosphorylation to facilitate the autophagic cell death pathways stimulated by BPA. Hence, our findings demonstrate that the pharmacological effect of astaxanthin on abnormal dermal ERK activation is mediated by its antioxidative potential against BPA. The important finding here is that astaxanthin has an inhibitory effect on the phosphorylation of NF-κB mediated by ERK activation to block autophagic cell death as induced by BPA. NF-κB is a multifaceted transcriptional regulator of the fundamental aspects of autophagy, apoptosis, and inflammation [29,30,31]. Earlier works have shown that NF-κB stimulated by exogenous factors is segregated from the cytoplasmic complex where it translocates into the nucleus after the degradation of the IκBα [32,33,34]. Considering the functional role of ERK in the phosphorylation of NF-κB, previous reports suggested that phosphorylated ERK1/2 could move into the nucleus, where it activates various transcriptional factors [35]. Hence, it is plausible that astaxanthin has a functional role in abrogating the NF-κB pathway through the suppression of ERK phosphorylation. These results indicate that NF-κB is a core transcription factor of the BPA signaling pathway that also regulates the autophagic cell death as inhibited by astaxanthin.

In support of these findings, our results revealed astaxanthin is able to regulate the transcriptional expression of the autophagy genes (LC3-II, Beclin-1, Atg12, Atg14) via the inhibition of NF-κB activity in BPA-treated NHDF. As central elements enhancing the autophagic process, Beclin-1 and Atg14 has been shown to regulate the localization of autophagic proteins, triggering the formation of the pre-autophagosomal structure [36,37], while Atg12 and LC3-II has been demonstrated to play an essential role in autophagy as it relates to biogenesis and in the transport of autophagosomes by functioning during the elongation of the phagophore double-layer membrane [38,39,40,41]. Indeed, earlier work showed the transcription factor NF-κB directly binds to the promoters of Beclin-1 and LC3-II and upregulates its mRNA and protein levels, leading to the autophagic process [36,42]. Thus, our findings are consistent with the idea that astaxanthin inhibits the transcriptional regulation of NF-κB for close working together with the cellular autophagic machinery to protect against oxidative dermal damage caused by BPA exposure. Intriguingly, the inhibition of the autophagic process in NHDF reduces the level of apoptotic cell death, suggesting the involvement of the autophagic machinery coupled with cell death as elicited by BPA. This result is supported by previous findings showing that the cleavage of Beclin-1 mediated by the caspase activation promotes crosstalk between autophagy and apoptosis [43]. Taken together, the biological activities of astaxanthin obtained from this study strongly indicate that astaxanthin may be a viable candidate as a potent anti-oxidative agent to inhibit autophagic cell death induced by BPA in dermal fibroblasts.

## 5. Conclusions

Our findings indicate that astaxanthin abrogates the autophagic cell death initiated by BPA and that the ROS production coupled with activation of ERK and NF-κB is a necessary step in the promotion of autophagy responsible for the apoptosis in the human dermal fibroblasts. This suggests that autophagic cell death is a predominant mechanism for controlling cell viability in the promoting of skin dermal damages induced by BPA. Thus, studies regarding the ROS/ERK/NF-κB signaling pathway regulated by astaxanthin during the BPA exposure are likely to be critical to develop the pharmacological and cosmetic agents against the skin dermal damages.

## Data Availability

Data is contained within the article and Supplementary Materials.

## Supplementary Materials

The following are available online at https://www.mdpi.com/article/10.3390/antiox10081273/s1, Table S1: PCR primer sequences, Figure S1: The role of astaxanthin on the level of autophagy-related proteins in BPA-treated NHDF.

## Abbreviations

BPA: bisphenol A; CM-H_2_DCFDA: 2′,7′-dichlorofluorescein diacetate; ERK: extracellular signal-regulated kinase; LC3-II: microtubule-associated proteins light chain-II; MAPKs: mitogen-activated protein kinases; NF-κB: nuclear factor-kappa B; NHDF: normal human dermal fibroblasts; ROS: reactive oxygen species.

## References

[1] Zulkifli S., Rahman A.A., Kadir S., Nor N.S.M. (2021). Bisphenol A and its effects on the systemic organs of children. Eur. J. Pediatr..

[2] Bhandari R.K., Deem S.L., Holliday D.K., Jandegian C.M., Kassotis C.D., Nagel S.C., Tillitt D.E., Vom Saal F.S., Rosenfeld C.S. (2015). Effects of the environmental estrogenic contaminants bisphenol A and 17α-ethinyl estradiol on sexual development and adult behaviors in aquatic wildlife species. Gen. Comp. Endocrinol..

[3] Mahemuti L., Chen Q., Coughlan M.C., Qiao C., Chepelev N.L., Florian M., Dong D., Woodworth R.G., Yan J., Cao X.L. (2018). Bisphenol A induces DSB-ATM-p53 signaling leading to cell cycle arrest, senescence, autophagy, stress response, and estrogen release in human fetal lung fibroblasts. Arch. Toxicol..

[4] Dyring-Andersen B., Lovendorf M.B., Coscia F., Santos A., Moller L.B.P., Colaco A.R., Niu L., Bzorek M., Doll S., Andersen J.L. (2020). Spatially and cell-type resolved quantitative proteomic atlas of healthy human skin. Nat. Commun..

[5] Sorrell J.M., Arnold I.C. (2004). Fibroblast heterogeneity: More than skin deep. J. Cell Sci..

[6] Bogaki T., Mitani K., Oura Y., Ozeki K. (2017). Effects of ethyl-α-D-glucoside on human dermal fibroblasts. Biosci. Biotechnol. Biochem..

[7] Aldag C., Nogueira Teixeira D., Leventhal P.S. (2016). Skin rejuvenation using cosmetic products containing growth factors, cytokines, and matrikines: A review of the literature. Clin. Cosmet Investig. Dermatol..

[8] Zalko D., Jacques C., Duplan H., Bruel S., Perdu E. (2011). Viable skin efficiently absorbs and metabolizes bisphenol A. Chemosphere.

[9] Lv Y., Lu S., Dai Y., Rui C., Wang Y., Zhou Y., Li Y., Pang Q., Fan R. (2017). Higher dermal exposure of cashiers to BPA and its association with DNA oxidative damage. Environ. Int..

[10] Mizushima N. (2007). Autophagy: Process and function. Genes Dev..

[11] Gu Y., Han J., Jiang C., Zhang Y. (2020). Biomarkers, oxidative stress and autophagy in skin aging. Ageing Res. Rev..

[12] Liu Y., Levine B. (2015). Autosis and autophagic cell death: The dark side of autophagy. Cell Death Differ..

[13] Kishimoto Y., Yoshida H., Kondo K. (2016). Potential Anti-Atherosclerotic Properties of Astaxanthin. Mar. Drugs..

[14] Sztretye M., Dienes B., Gonczi M., Czirjak T., Csernoch L., Dux L., Szentesi P., Keller-Pinter A. (2019). Astaxanthin: A Potential Mitochondrial-Targeted Antioxidant Treatment in Diseases and with Aging. Oxid. Med. Cell. Longev..

[15] Song X., Wang B., Lin S., Jing L., Mao C., Xu P., Lv C., Liu W., Zuo J. (2014). Astaxanthin inhibits apoptosis in alveolar epithelial cells type II in vivo and in vitro through the ROS-dependent mitochondrial signalling pathway. J. Cell. Mol. Med..

[16] Zuluaga M., Gueguen V., Letourneur D., Pavon-Djavid G. (2018). Astaxanthin-antioxidant impact on excessive Reactive Oxygen Species generation induced by ischemia and reperfusion injury. Chem. Biol. Interact..

[17] Liu X., Shibata T., Hisaka S., Osawa T. (2009). Astaxanthin inhibits reactive oxygen species-mediated cellular toxicity in dopaminergic SH-SY5Y cells via mitochondria-targeted protective mechanism. Brain Res..

[18] Kim J.H., Choi W., Lee J.H., Jeon S.J., Choi Y.H., Kim B.W., Chang H.I., Nam S.W. (2009). Astaxanthin inhibits H_2_O_2_-mediated apoptotic cell death in mouse neural progenitor cells via modulation of p38 and MEK signaling pathways. J. Microbiol. Biotechnol..

[19] Xue X.L., Han X.D., Li Y., Chu X.F., Miao W.M., Zhang J.L., Fan S.J. (2017). Astaxanthin attenuates total body irradiation-induced hematopoietic system injury in mice via inhibition of oxidative stress and apoptosis. Stem Cell Res. Ther..

[20] Liu Y., Yang L., Guo Y., Zhang T., Qiao X., Wang J., Xu J., Xue C. (2020). Hydrophilic Astaxanthin: PEGylated Astaxanthin Fights Diabetes by Enhancing the Solubility and Oral Absorbability. J. Agric. Food Chem..

[21] Davinelli S., Nielsen M.E., Scapagnini G. (2018). Astaxanthin in Skin Health, Repair, and Disease: A Comprehensive Review. Nutrients.

[22] Fang Q., Guo S., Zhou H., Han R., Wu P., Han C. (2017). Astaxanthin protects against early burn-wound progression in rats by attenuating oxidative stress-induced inflammation and mitochondria-related apoptosis. Sci. Rep..

[23] Kim D.W., Choi C.H., Park J.P., Lee S.J. (2020). Nanospheres Loaded with Curcumin Improve the Bioactivity of Umbilical Cord Blood-Mesenchymal Stem Cells via c-Src Activation During the Skin Wound Healing Process. Cells.

[24] Kim J.Y., Lee Y.M., Kim D.W., Min T., Lee S.J. (2020). Nanosphere Loaded with Curcumin Inhibits the Gastrointestinal Cell Death Signaling Pathway Induced by the Foodborne Pathogen *Vibrio Vulnificus*. Cells.

[25] Bryk D., Olejarz W., Zapolska-Downar D. (2014). Mitogen-activated protein kinases in atherosclerosis. Postepy Hig. Med. Dosw. (Online).

[26] Wu D., Luo N., Wang L., Zhao Z., Bu H., Xu G., Yan Y., Che X., Jiao Z., Zhao T. (2017). Hydrogen sulfide ameliorates chronic renal failure in rats by inhibiting apoptosis and inflammation through ROS/MAPK and NF-kappaB signaling pathways. Sci. Rep..

[27] Singh K.N., Patil S., Barkate H. (2020). Protective effects of astaxanthin on skin: Recent scientific evidence, possible mechanisms, and potential indications. J. Cosmet Dermatol..

[28] Wang C., Li Z., Han H., Luo G., Zhou B., Wang S., Wang J. (2016). Impairment of object recognition memory by maternal bisphenol A exposure is associated with inhibition of Akt and ERK/CREB/BDNF pathway in the male offspring hippocampus. Toxicology.

[29] Kumar A., Takada Y., Boriek A.M., Aggarwal B.B. (2004). Nuclear factor-kappaB: Its role in health and disease. J. Mol. Med..

[30] Luo J.L., Kamata H., Karin M. (2005). IKK/NF-κB signaling: Balancing life and death-a new approach to cancer therapy. J. Clin. Investig..

[31] Park A., Koh H.C. (2019). NF-κB/mTOR-mediated autophagy can regulate diquat-induced apoptosis. Arch. Toxicol..

[32] An Y., Zhang H., Wang C., Jiao F., Xu H., Wang X., Luan W., Ma F., Ni L., Tang X. (2019). Activation of ROS/MAPKs/NF-κB/NLRP3 and inhibition of efferocytosis in osteoclast-mediated diabetic osteoporosis. FASEB J..

[33] Tak P.P., Firestein G.S. (2001). NF-κB: A key role in inflammatory diseases. J. Clin. Investig..

[34] Lawrence T., Bebien M., Liu G.Y., Nizet V., Karin M. (2005). IKKα limits macrophage NF-κB activation and contributes to the resolution of inflammation. Nature.

[35] Lidke D.S., Huang F., Post J.N., Rieger B., Wilsbacher J., Thomas J.L., Pouyssegur J., Jovin T.M., Lenormand P. (2010). ERK nuclear translocation is dimerization-independent but controlled by the rate of phosphorylation. J. Biol. Chem..

[36] Kang R., Zeh H.J., Lotze M.T., Tang D. (2011). The Beclin 1 network regulates autophagy and apoptosis. Cell Death Differ..

[37] Obara K., Ohsumi Y. (2011). Atg14: A key player in orchestrating autophagy. Int. J. Cell Biol..

[38] Kabeya Y., Mizushima N., Ueno T., Yamamoto A., Kirisako T., Noda T., Kominami E., Ohsumi Y., Yoshimori T. (2000). LC3, a mammalian homologue of yeast Apg8p, is localized in autophagosome membranes after processing. EMBO J..

[39] Shpilka T., Weidberg H., Pietrokovski S., Elazar Z. (2011). Atg8: An autophagy-related ubiquitin-like protein family. Genome Biol..

[40] Lang T., Schaeffeler E., Bernreuther D., Bredschneider M., Wolf D.H., Thumm M. (1998). Aut2p and Aut7p, two novel microtubule-associated proteins are essential for delivery of autophagic vesicles to the vacuole. EMBO J..

[41] Otomo C., Metlagel Z., Takaesu G., Otomo T. (2013). Structure of the human ATG12~ATG5 conjugate required for LC3 lipidation in autophagy. Nat. Struct. Mol. Biol..

[42] Copetti T., Bertoli C., Dalla E., Demarchi F., Schneider C. (2009). p65/RelA modulates BECN1 transcription and autophagy. Mol. Cell Biol..

[43] Djavaheri-Mergny M., Maiuri M.C., Kroemer G. (2010). Cross talk between apoptosis and autophagy by caspase-mediated cleavage of Beclin 1. Oncogene.

