# Supplementary material for: Astaxanthin Inhibits Autophagic Cell Death Induced by Bisphenol A in Human Dermal Fibroblasts
Source: Antioxidants (Basel). 2021 Aug 11;10(8):1273. doi: 10.3390/antiox10081273 (PMC8389241; doi:10.3390/antiox10081273)
Supplement: Supplementary file 1 [file antioxidants-10-01273-s001.zip › antioxidants-1305170-supplementary.pdf]

Supplementary data

Astaxanthin Inhibits Autophagic Cell Death Induced by Bisphenol A in Human Dermal Fibroblasts

Seong-Ryeong Lim [1], Do-Wan Kim [1], Junghee Sung [2], Tae Hoon Kim [3],
Chang-Hyung Choi [4,*] and Sei-Jung Lee [1,*]

[1] *Department of Pharmaceutical Engineering, Daegu Haany University, Gyeongsan 38610, South Korea*

[2] *Research Center, Reanzen Co. Ltd., Anyang 14056, South Korea*

[3] *FoodyWorm Inc., Yancheongsongdae-gil 10, Ochang-eup, Cheongwon-gu, Choenju-si 28118, South Korea;*

[4] *Division of Cosmetic Science and Technology, Daegu Haany University, Gyeongsan 38610, South Korea*

*** Corresponding authors**: Sei-Jung Lee Ph.D. and Chang-Hyung Choi, Ph.D.

Department of Pharmaceutical Engineering, Daegu Haany University

Gyeongsan 38610, South Korea

E-mail: sjlee@dhu.ac.kr; Tel: 82-54-819-1806; Fax: 82-54-819-1406

Table S1. PCR primer sequences

Gene	Identification	Primer sequence, 5`-3`
ULK1	Forward	CAGAACTACCAGCGCATTGA
	Reverse	TCCACCCAGAGACATCTTCC
ULK2	Forward	CTCCTCAGGTTCTCCAGTGC
	Reverse	TTGGTGGGAGAAGTTCCAAG
Vps34	Forward	ATGTGTATGGTCCCGGAAAA
	Reverse	TGTCGATGAGCTTTGGTGAG
Atg14	Forward	TCACCATCCAGGAACTCACA
	Reverse	TTCAGTCTTCGGCTGAGGTT
Beclin-1	Forward	GCCCTTTGAAGTACGAGCAG
	Reverse	GAGATCATCCCACCTGCACT
Atg5	Forward	CGGGAACACCAAGTTTCACT
	Reverse	TCTGGGGAGACATCCGTAAG
Atg12	Forward	TGGGATTGCAAAATGACAGA
	Reverse	TTCCCCATCTTCAGGATCAA
Atg16L1	Forward	GTCTTCGATGCACATGATGG
	Reverse	GATTCGGCTTGCAAAATCAT
LC3-II	Forward	AGCAGCATCCAACCAAAATC
	Reverse	CTGTGTCCGTTCACCAACAG
Rab7	Forward	CTGACCAAGGAGGTGATGGT
	Reverse	CTGGCCTGGATGAGAAACTC
FYCO1	Forward	GGAGCTAGGAGCAGCAGAGA
	Reverse	CGCATCACTGGGAATAGGTT
LAMP1	Forward	CTTCAGCAGGGGAGAGACAC
	Reverse	TGTTGGGGTTGATGTTGAGA
LAMP2	Forward	GGTTAATGGCTCCGTTTTCA
	Reverse	ATGGGCACAAGGAAGTTGTC
β-actin	Forward	AACCGCGAGAAGATGACCCAGATCATGTTT
	Reverse	AGCAGCCGTGGCCATCTCTTGCTCGAAGTC

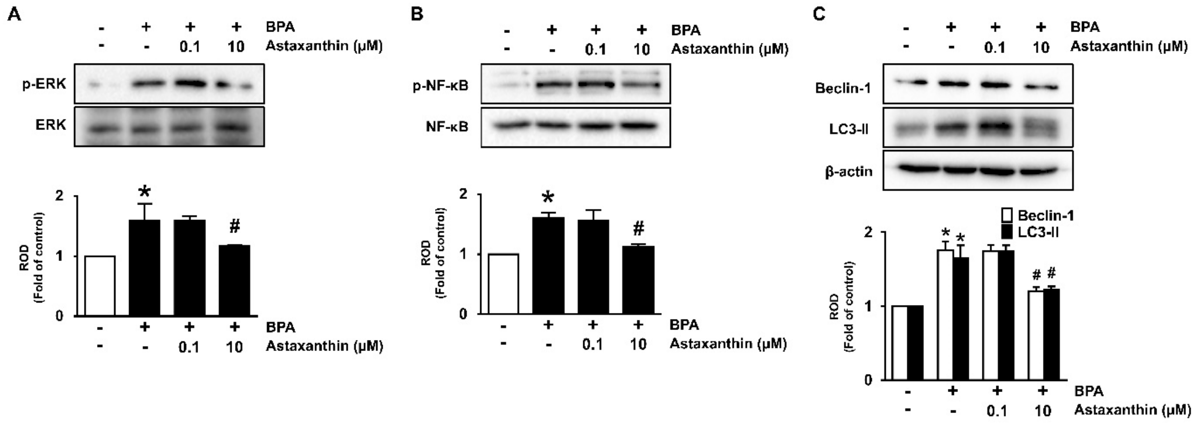

Figure S1. Dose-dependent responses of astaxanthin on the level of autophagy-related proteins in BPA-treated NHDF are shown. The effects of astaxanthin (0.1 and 10 μM) on the phosphorylation of ERK **(A)** and NF-κB **(B)**, and the expression of Beclin-1 and LC3-II **(C)** are shown. * $p \leq 0.05$ versus control, # $p \leq 0.001$ versus BPA alone. n = 3. ROD, relative optical density.
